# Supplementary material for: Purified regenerating retinal neurons reveal regulatory role of DNA methylation-mediated Na+/K+-ATPase in murine axon regeneration
Source: Commun Biol. 2023 Jan 30;6:120. doi: 10.1038/s42003-023-04463-4 (PMC9886953; doi:10.1038/s42003-023-04463-4)
Supplement: Supplementary file 2 — Supplementary Information [file 42003_2023_4463_MOESM2_ESM.pdf]

Supplementary Information

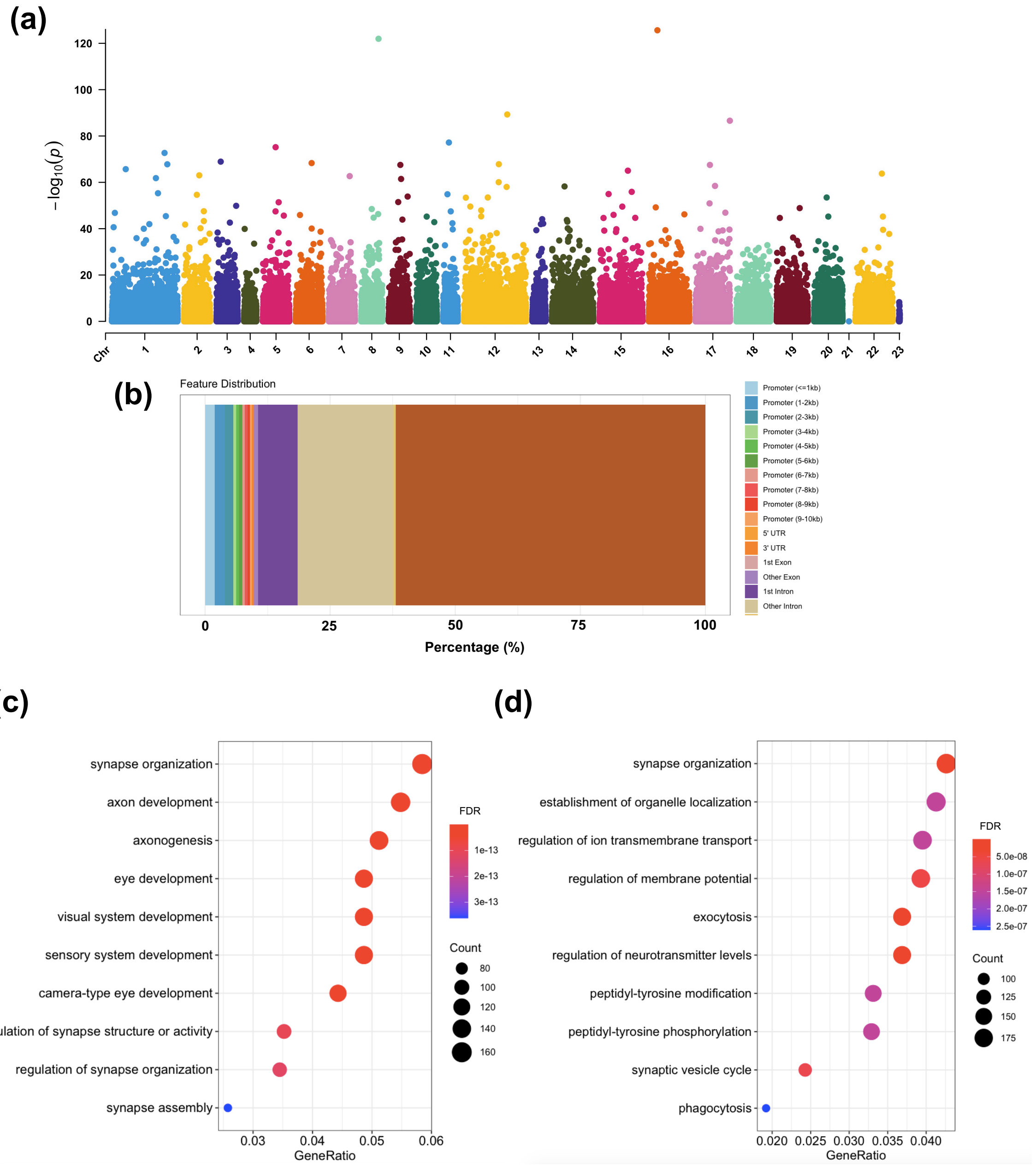

Supplementary Figure 1: Distribution of DMLs. (a) A Manhattan plot shows the genome-wide

distribution across all chromosomes (x-axis) of all CpGs tested for differential methylation (y-axis) between injured/regenerated (IR) (*N* = 12 retinas) and injured/non-regenerated (INR) (*N* = 12 retinas) RGC groups. (b) A modified bar plot shows the distribution of DMLs as annotated to standard genomic features. Varying genomic features are color-coordinated. The proportion of DMRs associated with each genomic feature are depicted by the width of each bar (x-axis). (c-d) A dot plot shows the top ten ontological terms of biological processes (y-axis) identified from genes linked to regeneration-associated hypermethylated and hypomethylated DMLs, respectively. The GeneRatio (x-axis) depicts the ratio of DML-associated genes over the number of genes in the entire genome that are associated with each term displayed. The size of the dots represents the relative number of DML-associated genes for each term. Dots are colored based on FDR *P*-value.

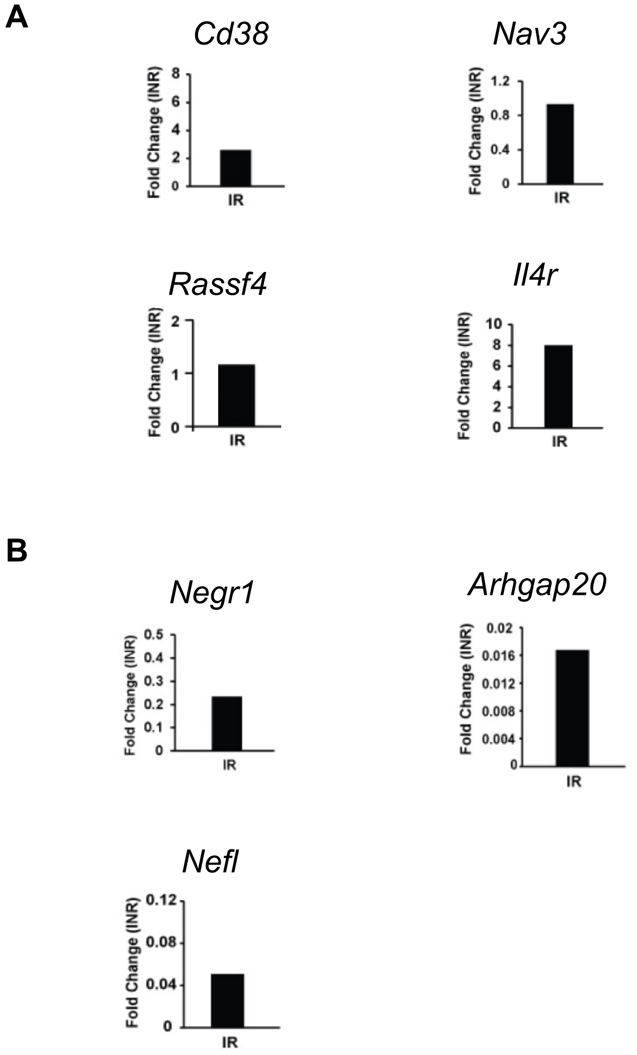

Supplementary Figure 2: Quantitative-RT-PCR results of regeneration-associated genes harboring DMLs. (a) Quantitative-RT-PCR results of four representative regeneration-associated genes containing DMLs that displayed significantly induced expression in injured/regenerated (IR) RGCs (x-axis) compared to injured/non-regenerated (INR) RGCs (fold-change; y-axis). (b)

Quantitative-RT-PCR results of three representative regeneration-associated genes containing DMLs that displayed significantly reduced expression in IR RGCs (x-axis) compared to INR RGCs (fold-change; y-axis).

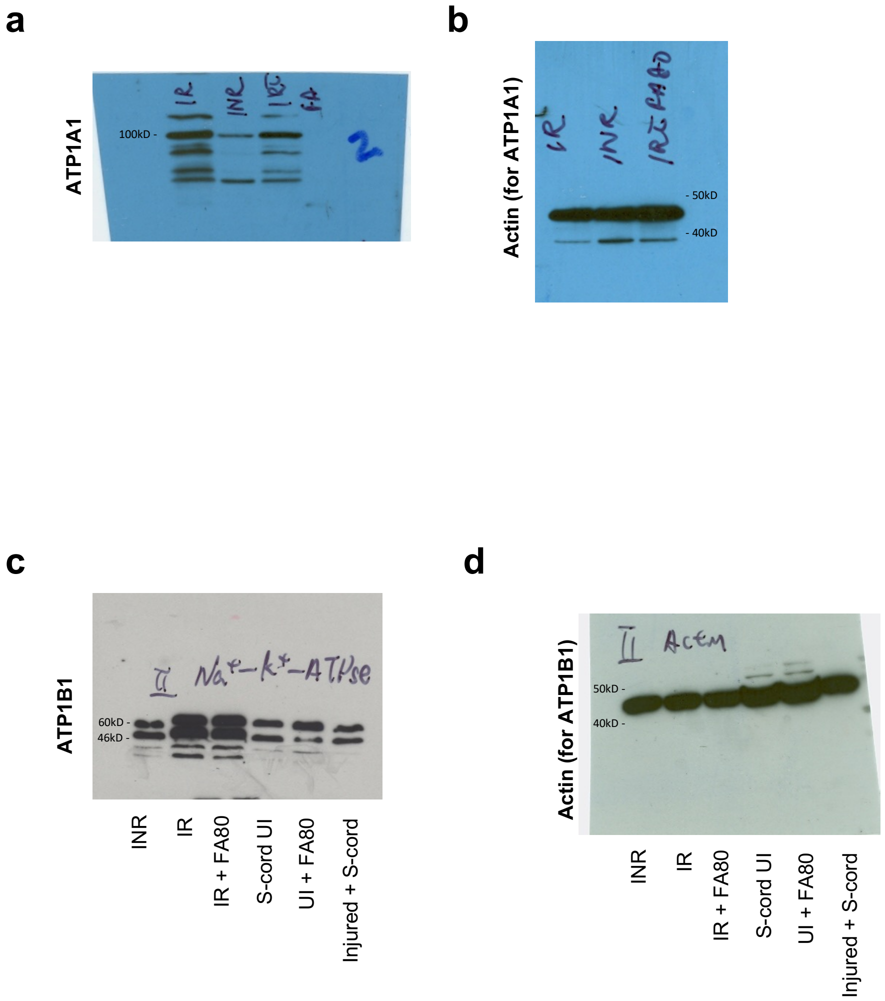
a
ATP1A1
100kD -
b
Actin (for ATP1A1)
- 50kD
- 40kD
c
ATP1B1
60kD -
46kD -
INR
IR
IR + FA80
S-cord UI
UI + FA80
Injured + S-cord
d
Actin (for ATP1B1)
50kD -
40kD -
INR
IR
IR + FA80
S-cord UI
UI + FA80
Injured + S-cord

Supplementary Figure 3: Full immunoblots of ATP1A1 and ATP1B1. (a) A representative immunoblot staining for ATP1A1 is depicted. The first lane represents protein extracted from an injured/regenerated (IR) sample, the second lane represents extracts from an injured/non-regenerated (INR) sample, the third lane represents extracts used for other experiments not reported in this manuscript. (b) A representative immunoblot staining for actin for the ATP1A1 immunoblot. Samples run in each lane parallel those in (a). (c) A representative immunoblot staining for ATP1B1 is depicted. The first lane represents protein extracted from an injured/non-regenerated (INR) sample, the second lane represents extracts from an injured/regenerated (IR) sample, the third, fourth, fifth, and sixth lanes represent samples used for other experiments not reported in this manuscript. (d) A representative immunoblot staining for actin for the ATP1B1 immunoblot. Samples run in each lane parallel those in (c).

	Raw reads (million)	Reads passing QC (I) (million)	Aligned reads (I) (million)	Reads passing QC (II) (million)	Aligned reads (II) (million)	Total alignment efficiency (%)	Number of CpGs >=10x (million)
IR.1	882.6	862.9	495.2	294.9	100.1	69	13.1
IR.2	759.2	741.6	420.9	258.4	88.5	68.7	11.3
IR.3	547.6	533.3	287.6	171.3	43.6	62.1	6.7
INR.1	843.4	822.4	454.2	283.6	104.2	67.9	17.6
INR.2	895.6	874.5	452.4	339.6	127.9	66.4	18
INR.3	722.4	711.5	309.6	330.2	125	61.1	16.6
UI.1	714.8	700.4	384.3	243.6	91.5	67.9	17
UI.2	894.2	862.3	485.2	240.4	77.4	65.2	16.5
UI.3	788.2	773.6	420	284	107.6	68.2	18.1
E.1	749	738.1	419.2	268.4	104.7	71	17.2
E.2	793.6	780	420.1	296.9	117	68.9	18.2
E.3	754.4	741.7	393.8	284.8	107.6	67.6	18.1

Supplementary Table 1: Summary of whole-genome methylation sequencing analysis.

Transcription factor (hyper-DMRs)	Q-value		Transcription factor (hypo-DMRs)	Q-value
DLX5	0		ELF5	0
DLX2	0		PU.1	0
Lhx3	0		Elf4	0
LHX9	0		ELF3	0
Lhx2	0		EHF	0
Lhx1	0		IRF8	0
DLX1	0		ETV1	0
Dlx3	0.0001		ELF1	0
En1	0.002		ETS1	0
Phox2a	0.0022		GABPA	0
Nkx6.1	0.0022		SpiB	0
GSC	0.0022		Fli1	0
Isl1	0.0055		EWS	0.0001
Brn1	0.0058		Etv2	0.0006
Prop1	0.0067		IRF1	0.0009
			ERG	0.0013
			ETV4	0.0022

Supplementary Table 2: Significantly enriched transcription factors in DMR sequences.
