# Supplementary material for: Purified regenerating retinal neurons reveal regulatory role of DNA methylation-mediated Na+/K+-ATPase in murine axon regeneration
Source: Commun Biol. 2023 Jan 30;6:120. doi: 10.1038/s42003-023-04463-4 (PMC9886953; doi:10.1038/s42003-023-04463-4)
Supplement: Supplementary file 3 — Description of Additional Supplementary Files [file 42003_2023_4463_MOESM3_ESM.pdf]

Description of Additional Supplementary Files

File name: Supplementary Data 1
Description: DML results.
