# Supplementary material for: Purified regenerating retinal neurons reveal regulatory role of DNA methylation-mediated Na+/K+-ATPase in murine axon regeneration
Source: Commun Biol. 2023 Jan 30;6:120. doi: 10.1038/s42003-023-04463-4 (PMC9886953; doi:10.1038/s42003-023-04463-4)
Supplement: Supplementary file 5 — Reporting Summary [file 42003_2023_4463_MOESM5_ESM.pdf]

Corresponding author(s): Bermans Iskandar & Reid Alisch

Last updated by author(s): Oct 27, 2022

Reporting Summary

Nature Portfolio wishes to improve the reproducibility of the work that we publish. This form provides structure for consistency and transparency in reporting. For further information on Nature Portfolio policies, see our Editorial Policies and the Editorial Policy Checklist.

Statistics

For all statistical analyses, confirm that the following items are present in the figure legend, table legend, main text, or Methods section.

n/a	Confirmed	
☐	☒	The exact sample size (n) for each experimental group/condition, given as a discrete number and unit of measurement
☐	☒	A statement on whether measurements were taken from distinct samples or whether the same sample was measured repeatedly
☐	☒	The statistical test(s) used AND whether they are one- or two-sided<br>*Only common tests should be described solely by name; describe more complex techniques in the Methods section.*
☐	☒	A description of all covariates tested
☐	☒	A description of any assumptions or corrections, such as tests of normality and adjustment for multiple comparisons
☐	☒	A full description of the statistical parameters including central tendency (e.g. means) or other basic estimates (e.g. regression coefficient) AND variation (e.g. standard deviation) or associated estimates of uncertainty (e.g. confidence intervals)
☐	☒	For null hypothesis testing, the test statistic (e.g. F, t, r) with confidence intervals, effect sizes, degrees of freedom and P value noted<br>*Give P values as exact values whenever suitable.*
☒	☐	For Bayesian analysis, information on the choice of priors and Markov chain Monte Carlo settings
☒	☐	For hierarchical and complex designs, identification of the appropriate level for tests and full reporting of outcomes
☒	☐	Estimates of effect sizes (e.g. Cohen's d, Pearson's r), indicating how they were calculated

Our web collection on statistics for biologists contains articles on many of the points above.

Software and code

Policy information about availability of computer code

Data collection	Scripts and code used for the generation of data, results, and figures used in this manuscript have been deposited at https://github.com/andymadrid/RGC_DNAm.git. Video files can be viewed at https://uwmadison.box.com/s/mli3znh1av9coj67tw59si10se6v6ynr.
Data analysis	Scripts and code used for the generation of data, results, and figures used in this manuscript have been deposited at https://github.com/andymadrid/RGC_DNAm.git. Video files can be viewed at https://uwmadison.box.com/s/mli3znh1av9coj67tw59si10se6v6ynr.

For manuscripts utilizing custom algorithms or software that are central to the research but not yet described in published literature, software must be made available to editors and reviewers. We strongly encourage code deposition in a community repository (e.g. GitHub). See the Nature Portfolio guidelines for submitting code & software for further information.

Data

Policy information about availability of data

All manuscripts must include a data availability statement. This statement should provide the following information, where applicable:

- Accession codes, unique identifiers, or web links for publicly available datasets
- A description of any restrictions on data availability
- For clinical datasets or third party data, please ensure that the statement adheres to our policy

Scripts and code used for the generation of data, results, and figures used in this manuscript have been deposited at https://github.com/andymadrid/RGC_DNAm.git. Video files can be viewed at https://uwmadison.box.com/s/mli3znh1av9coj67tw59si10se6v6ynr.

Human research participants

Policy information about studies involving human research participants and Sex and Gender in Research.

Reporting on sex and gender	*Use the terms sex (biological attribute) and gender (shaped by social and cultural circumstances) carefully in order to avoid confusing both terms. Indicate if findings apply to only one sex or gender; describe whether sex and gender were considered in study design whether sex and/or gender was determined based on self-reporting or assigned and methods used. Provide in the source data disaggregated sex and gender data where this information has been collected, and consent has been obtained for sharing of individual-level data; provide overall numbers in this Reporting Summary. Please state if this information has not been collected. Report sex- and gender-based analyses where performed, justify reasons for lack of sex- and gender-based analysis.*
Population characteristics	*Describe the covariate-relevant population characteristics of the human research participants (e.g. age, genotypic information, past and current diagnosis and treatment categories). If you filled out the behavioural & social sciences study design questions and have nothing to add here, write "See above."*
Recruitment	*Describe how participants were recruited. Outline any potential self-selection bias or other biases that may be present and how these are likely to impact results.*
Ethics oversight	*Identify the organization(s) that approved the study protocol.*

Note that full information on the approval of the study protocol must also be provided in the manuscript.

Field-specific reporting

Please select the one below that is the best fit for your research. If you are not sure, read the appropriate sections before making your selection.

☒ Life sciences ☐ Behavioural & social sciences ☐ Ecological, evolutionary & environmental sciences

For a reference copy of the document with all sections, see nature.com/documents/nr-reporting-summary-flat.pdf

Life sciences study design

All studies must disclose on these points even when the disclosure is negative.

Sample size	N = 3 animals per group were used for this study. In large-scale omics-based methodologies, a minimum of three biological replicates are necessary to calculate variation, deviation, and dispersion amongst replicates.
Data exclusions	No samples were excluded from this study.
Replication	Three biological replicates were used to verify replication of differential methylation.
Randomization	Samples were not randomized as it was not relevant to data used here.
Blinding	Investigators were blinded during tissue acquisition and processing.

Reporting for specific materials, systems and methods

We require information from authors about some types of materials, experimental systems and methods used in many studies. Here, indicate whether each material, system or method listed is relevant to your study. If you are not sure if a list item applies to your research, read the appropriate section before selecting a response.

Materials & experimental systems

n/a	Involved in the study	
☐	☒	Antibodies
☒	☐	Eukaryotic cell lines
☒	☐	Palaeontology and archaeology
☐	☒	Animals and other organisms
☒	☐	Clinical data
☒	☐	Dual use research of concern

Methods

n/a	Involved in the study	
☒	☐	ChIP-seq
☐	☒	Flow cytometry
☒	☐	MRI-based neuroimaging

Antibodies

Antibodies used	cat#554898, BD Parmingen
Validation	Data were validated based on molecular size of bands during western blot analysis.

Animals and other research organisms

Policy information about studies involving animals; ARRIVE guidelines recommended for reporting animal research, and Sex and Gender in Research

Laboratory animals	Sprague-Dawley adult male rats
Wild animals	*Provide details on animals observed in or captured in the field; report species and age where possible. Describe how animals were caught and transported and what happened to captive animals after the study (if killed, explain why and describe method; if released, say where and when) OR state that the study did not involve wild animals.*
Reporting on sex	Only male animals were used in molecular studies here.
Field-collected samples	*For laboratory work with field-collected samples, describe all relevant parameters such as housing, maintenance, temperature, photoperiod and end-of-experiment protocol OR state that the study did not involve samples collected from the field.*
Ethics oversight	All animal housing and surgical procedures were conducted following the Guide for the Care and Use of Laboratory Animals and under conditions approved by The Research Animal Resources and Care Committee of the University of Wisconsin (M005286). Adult male Sprague Dawley rats were obtained from Harlan Laboratories, Madison, WI, USA. Rats were housed three per cage under standard 12 hours light/12 hours dark cycle, with food and water ad libitum. Heterozygous α-1 and α-2 mice were obtained from Jerry Lingrel, PhD (University of Cincinnati).

Note that full information on the approval of the study protocol must also be provided in the manuscript.

Flow Cytometry

Plots

Confirm that:

- [x] The axis labels state the marker and fluorochrome used (e.g. CD4-FITC).
- [x] The axis scales are clearly visible. Include numbers along axes only for bottom left plot of group (a 'group' is an analysis of identical markers).
- [ ] All plots are contour plots with outliers or pseudocolor plots.
- [ ] A numerical value for number of cells or percentage (with statistics) is provided.

Methodology

Sample preparation	Cells were initially gated based on relative size and granularity using forward-scatter and side-scatter to separate cells from debris, then single cells were gated on forward scatter area versus forward scatter height, followed by a second singlet gate using side scatter area versus side scatter height. Live single cells were then gated as DAPI negative. Finally, cells were gated into groups based on Thy-1 PE expression and Oregon Green fluorescence. Specifically, RGCs that regenerated axons (i.e., their axons reached the end of the sciatic nerve graft and were backfilled with Oregon Green) were Thy-1 positive/Oregon Green positive. Conversely, RGCs that did not regenerate axons after optic nerve transection, or those obtained from the embryonic or adult uninjured retinas, were Thy-1 PE positive/Oregon Green negative.
Instrument	Cytek Aurora Spectral Flow Cytometer
Software	FLOWJO
Cell population abundance	We selectively sorted Thy 1 PE-positive/Oregon Green-positive IR RGCs (389 ± 159 per retina, N = 12 retinas) and Thy-1 PE-positive/Oregon Green negative INR RGCs (117,567 ± 20,524 per retina, N = 12 retinas), along with purifying 150,000 ± 86,603 embryonic cells (UE) per retina (N = 12 retinas) and 166,667 ± 96,225 adult cells (UA).
Gating strategy	Cells were initially gated based on relative size and granularity using forward-scatter and side-scatter to separate cells from debris, then single cells were gated on forward scatter area versus forward scatter height, followed by a second singlet gate using side scatter area versus side scatter height. Live single cells were then gated as DAPI negative. Finally, cells were gated into groups based on Thy-1 PE expression and Oregon Green fluorescence. Specifically, RGCs that regenerated axons (i.e., their axons reached the end of the sciatic nerve graft and were backfilled with Oregon Green) were Thy-1 positive/Oregon Green positive. Conversely, RGCs that did not regenerate axons after optic nerve transection, or those obtained from the embryonic or adult uninjured retinas, were Thy-1 PE positive/Oregon Green negative.

- [ ] Tick this box to confirm that a figure exemplifying the gating strategy is provided in the Supplementary Information.
